# We Move in Mysterious Ways

**DOI:** 10.1371/journal.pbio.0020370

**Published:** 2004-09-21

A man in a suit and bowler hat walks awkwardly down the street, each convoluted step a labored movement. He lifts up one knee, then briefly stoops. Stepping forward, he swings the other leg out to the side then kicks high in the air. In this old Monty Python skit, the man works for the Ministry of Silly Walks. It's his job to walk this way. The rest of us, however, tend to stroll along—or throw baseballs, or lift coffee mugs—in a much more efficient manner.

There's a nearly infinite number of silly walks, throws, and lifts, but somehow people tend to settle on one best way of doing these things. However, scientists studying motor control have been hard pressed to figure out what exactly we're doing when we move. People may be striking a balance between sloth and speed: too slow and our throws lack oomph; too fast, and instead of dunking our donuts in our coffee, we dunk our whole fist. Or people might be minimizing some version of jerk—physicists' and engineers' term for changes in acceleration. (Roller coaster engineers, for example, balance jerk against speed and g's to keep the ride smooth and safe, but also fun.) But so far, such models that start by assuming people minimize error or jerk haven't allowed researchers to deduce what dictates how people move.

To help solve this recalcitrant problem, Konrad Körding and colleagues, as reported in PLoS Biology, took a page from economists, who have long used equations called utility functions that incorporate the costs and benefits of a situation. Say you like oranges better than apples, but oranges cost more. Given a certain budget for fruit, the utility function says how many of each you should buy. Similarly, Körding and colleagues observed people's preferred movements, then inferred an underlying utility function that presumably describes bias in the nervous system for different movements.

To see which movements people preferred, the researchers engaged people in a simple virtual reality system. The subjects moved a joystick that fought back: it was connected to a set of motors that produced varying forces—with a strong force for a short time, say, or a mild force for a longer time. Over and over, the subjects moved their cursors from one spot to another. After each pair of moves, the subjects then chose which of the two movements they found easier.

In this way, the researchers were able to rank a large set of different movements relative to each other by individuals' preferences. They found a surprising amount of agreement among the subjects on which movements were preferable. They also got a counterintuitive result: as the duration of the resistance got longer, people actually preferred stronger resistance. The researchers speculate that subjects didn't mind larger resistance when it acted over a longer period because the force takes longer to ramp up to its maximum value. Subjects would have more time to adjust—just as when someone gradually pushes into you, you can stay standing by leaning into them, whereas if they shove you with the same force it can knock you off balance.

By showing that utility functions can be of use not only in explaining the marketplace but also motor control, Körding and colleagues have added a new tool to biologists' repertoire. Though their approach hasn't closed the case on the mysteries of movement, it could help explain why we settle for a particular, non-silly walk.

